# Data Resource: the National Pupil Database (NPD)

**DOI:** 10.23889/ijpds.v4i1.1101

**Published:** 2019-03-20

**Authors:** Matthew Alexander Jay, Louise McGrath-Lone, Ruth Gilbert

**Affiliations:** 1 UCL Great Ormond Street Institute of Child Health, Population Policy & Practice Programme 30 Guilford Street London WC1N 1EH; 2 Great Ormond Street Hospital for Children NHS Foundation Trust, Department of Anaesthesia and Pain Medicine Great Ormond Street London WC1N 3JH; 3 The Rees Centre for Research in Fostering and Education University of Oxford 15 Norham Gardens Oxford OX2 6PY

## Abstract

**Introduction:**

The National Pupil Database (NPD) is a record-level administrative data resource curated by the UK government’s Department for Education that is used for funding purposes, school performance tables, policy making, and research.

**Processes:**

Data are sourced from schools, exam awarding bodies, and local authorities who collect data on an on-going basis and submit to the Department for Education either termly or yearly.

**Data contents:**

NPD contains child-level and school-level data on all pupils in state schools in England (6.6 million in the 2016/17 academic year). The primary module is the census, which has information on characteristics and school enrolment. Other modules include alternative provision, exam attainment, absence and exclusions. Data from children’s social care are also available on children referred for support and those who become looked after. Children’s records are linkable across different modules and across time using a nationally unique, anonymised child-level identifier. Linkage to external datasets has also been accomplished using child-level identifiers.

**Conclusions:**

The NPD is an especially valuable data resource for researchers interested in the educational experience and outcomes of children and young people in England. Although limited by the fact that children in private schools or who are home schooled are not included, it provides a near-complete picture of school trajectories and outcomes for the majority of children. Linkage to other datasets can enhance analyses and provide answers to questions that would otherwise be costly, time consuming and difficult to find

## Background

**Figure 1: Overview of datasets.**
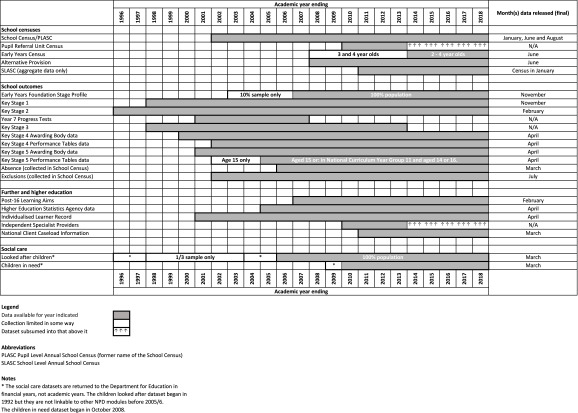


Educational attainment is associated with a range of health and social outcomes across life [1] and health states themselves can adversely affect educational outcomes [2]. Evaluation of children’s journeys through school and their ultimate attainment is therefore of key interest to a range of disciplines including education, social care, economics, epidemiology and others. Particularly from a longitudinal, life course perspective, high quality data collected at different points of life are necessary in order to investigate patterns of exposure and outcome over time. Here we present an overview of a rich and detailed, record-level, longitudinal, administrative data source—the National Pupil Database (NPD)—that can be used to follow all children in English state schools across their school careers and enumerate various outcomes including absences, exclusions, special educational needs, school changes and exam results [3]. Data from local authority children’s social care departments on children looked after and children in need are also available. The NPD is curated by the United Kingdom government’s Department for Education (DfE) pursuant to statutory duties and it is used for funding purposes, school performance tables, policy making and research [4,5]. An overview of the different modules that comprise the NPD is given in Figure 1 and these are described in more detail in the present manuscript.

## Data creation processes

### Source and linkage

NPD data are sourced from a number of organisations including schools, local authorities and exam awarding bodies, which have statutory duties to provide data for the NPD. Data are initially collected on organisations’ own management information systems on an event-driven basis (i.e. records are updated contemporaneously to the events documented). Schools and local authorities transfer data directly to the DfE using a centralised on-line portal (technical guidance on collection and submission is available [6]) and data from exam bodies are submitted to DfE via a private contractor [4]. Different modules of the NPD are collected at different intervals. The right-most column in Figure 1 shows the months in which data are made available to researchers.

### Data validation and quality

Data are subject to automatic quality control checks on submission by the DfE’s system. These include checks for totals that do not sum, values that may be out of range, and potential errors such as a school having no children with special educational needs. The onus is on the body submitting the data to check and correct them before the submission is approved. A full list of validation rules is publicly available [6].

## Data contents

### Overview

The NPD consists of several modules. The data source for pupil enrolments are the censuses, the primary one of which is the School Census, which replaced the Pupil Level Annual School Census in 2006 for secondary schools and 2007 for primary, nursery and special schools. Carried out termly (January, May and October), the School Census consists of data on pupils in state nurseries, primary, secondary and special schools. The Early Years Census includes pre-schoolers who are taking up a free pre-school place and the Alternative Provision Census provides a snapshot of children who are unable to attend school for reasons such as being excluded or on medical grounds. Data are also available on a range of outcomes in other modules, namely attainment at Key Stages 1 to 5 (at certain points across ages 7 to 18), absences and exclusions, as well as on further and higher education including Post-16 Learning Aims of children in schools with sixth forms. NPD data can also be linked by the DfE to information on university education collected by the Higher Education Statistics Agency (all UK Higher Education institutions) and in the Individualised Learner Record (further education and apprenticeships or work-based learning). [7] Details of all available data items can be found in the DfE data tables, which are currently available by contacting the DfE [8].

Each variable is assigned a sensitivity level from A to E and an identifiability risk from 1 to 5; they were previously assigned Tiers from 1 to 4 (Table 1). For some variables, less identifiable versions are available. For example, date of birth is identifiable 4 (sensitivity E) but year and month of birth and year at the start of the academic year are identifiable 5 (sensitivity E).

**Table 1: Validation of child health groupings:** NPD National Pupil Database; DfE Department for Education; GDPR General Data Protection Regulation; SEN Special Educational Needs

Tiers (old system)	Identifiability risk	Sensitivity

Tier 1 (highly sensitivity child-level that requires justification as to why it is needed and why Tier 2 is not sufficient) Tier 2 (highly sensitive that requires justification as to why the data item is needed) Tier 3 (school-level data) Tier 4 (child-level as standard in NPD extracts)	1 (instant identifier) 2 (meaningful identifier) 3 (meaningless identifier) 4 (non-identifier that has a high risk of identification associated with it) 5 (other)	A (not used outside DfE) B (highly sensitive) C (sensitive not captured as part of a special GDPR category) D (sensitive captured as part of a special GDPR category, e.g. SEN) E (other)

Children’s data are linkable over time (e.g. across different census years) and across different modules using the Pupil Matching Reference (PMR)—a nationally unique and anonymised child-level identifier. The PMR is based on the Unique Pupil Number, which is usually assigned to a child by local authorities on starting state school, though this can be earlier, e.g. where a child has an Education, Health and Care Plan [9]. The Unique Pupil Number is recorded in NPD but is not routinely disclosed.

### The censuses

The NPD censuses provide data on the characteristics of all children enrolled in state schools in England each year. A summary of measures available in the NPD censuses is given in Table 2.

**Table 2: Key variables in National Pupil Database censuses:** SC School Census; PRU Pupil Referral Unit Census; EY Early Years Census; AP Alternative Provision; LA local authority) There have been changes in some of these variables over time and details may vary for different censuses. * Includes SC, PRU, EY and AP. Identifiability and sensitivity levels are explained in Table 1.

Variable	Census	Identifiability	Sensitivity	Details / less sensitive alternatives

**Child-level** Useful for linkage				
Pupil matching reference	All*	3	E	Anonymised child-level identifier based on Unique Pupil Number with salience only to NPD. A non-anonymised version and the Unique Pupil Number are available (both identifiability 2).
Gender	All*	5	E	
Date of birth	All*	4	E	Lower tier age variables include month and year of birth, age at start of academic year and month part of age at start of academic year (all identifiability 5).
Names	All*	1	E	Forenames, surnames, middle names and former surname.
Ethnicity	All*	4	D	Also available as ethnic groups major and minor.
Postcode	All*	4	E	Census output area and lower-layer super output areas also available (same sensitivity and identifiability).
**Child-level** Other key variables				
Language	SC, PRU	4	D	Also available as first language, except in PRUs (identifiability 5), and language group major and minor (identifiability 5).
Free school meals	SC, PRU, AP	5	C	Whether child recorded as eligible for free school meals. Available as currently eligible and eligible over the past 3 or 6 years or ever.
Special educational needs	All*	5	D	Available are primary and secondary need type. Special educational needs provision (i.e. whether the child receives support, a statement or an Education, Health and Care Plan) is also available.
Absences	SC (supplied separately)	4-5	C-E	Data on the number of possible sessions per term, the number of authorised absences and the number of unauthorised absences as well as reasons for absences.
Exclusions	SC (supplied separately)	4-5	C-E	Data on the number of sessions excluded from and the category of exclusions (fixed or permanent), as well as reasons for exclusions.
Dates of joining and leaving	SC, PRU	5	E	Dates that the pupil joined and left the school.
**School-level**				
Unique reference number	SC, PRU, EY	4	E	DfE school unique reference number.
Establishment code	SC, PRU	5	E	Establishment code assigned by the DfE.
LA-establishment code	SC, PRU	4	E	Local authority code and establishment code joined together.
LA	All*	5	E	Code identifying the local authority that the school reports to.
**Area-based**				
Output areas	SC, PRU	4	E	Based on the child’s postcode, either the 2001 or 2011 versions depending on academic year. Census output areas and lower-layer super output areas available.
Home LA	SC, PRU	5	E	The local authority in whose area the child lives.

Particularly important in health and educational contexts is whether the child receives support for special educational needs and the type of needs that the child has. The NPD includes information on the primary type of special educational needs a pupil has, as well as the type of in-school support they receive as a result. A separate disability variable (‘type of disability’) was available for the 2010/11 and 2011/12 academic years. Information on whether the child is recorded as eligible for receiving free school meals is also captured and this is available as either current eligibility or being recorded as eligible in the past 3 or 6 years or ever. Eligibility for free school meals is based on the parents’ receiving certain means-tested benefits [10] and can therefore be used as a crude socioeconomic position indicator.

### Attainment

Table 3 provides a summary of attainment data at the Early Years Foundation Stage Profile (ages 3 to 5) and Key Stages 1 to 5 (ages 7 to 18). Note that between the 2002/3 and 2005/6 academic years, data only on a 10% sample of children undergoing the early years foundation stage were collected; from 2006/7, all children in receipt of a government funded early education place are included. Children’s data at each stage can be linked using the PMR thus facilitating the construction of ‘value added’ models (i.e. adjustment for prior attainment). The rich level of data available at Key Stage 4, in particular, also allows for the analysis of attainment according to different specifications (e.g. total General Certificate of Secondary Education (GCSE) points versus meeting a given threshold such as five passes at A* - C) [11,12]. Researchers will have to carefully consider how to handle data at each Key Stage, particularly as: attainment scales for exams have changed over time; exams at different stages are measured on different scales; children do not always sit all (or any) exams; and that children will sit a different number of exams [13].

**Table 3: Summary of National Pupil Database attainment data:** EYFSP Early years foundation stage profile; KS Key Stage; GCSE General Certificate of Secondary Education; GNVQ General National Vocational Qualification; GCE General Certificate of Education

Stage	Ages (School years)	Details

EYFSP	3-5 (Early years / reception)	There are 13 subscales scored 0-9, which measure communication; physical development; personal, social and emotional development; literacy; mathematics; understanding the world; and expressive arts and designs. Assessment is by the class teacher on the basis of classroom observations. Data are available on the overall score, each subscale score and each element that makes up the scores.
KS1	5-7 (Years 1-2)	Tests at Key Stage 1 include teacher-assessed English reading, spelling, punctuation and grammar, science and mathematics. Data on whether the child underwent each test and the level achieved are available.
KS2	7-11 (Years 3-6)	Children sit tests in the same subjects at the end of Key Stage 2 as at Key Stage 1. The level achieved as well as the actual marks are stored in NPD.
KS3	11-14 (Years 7-9)	Key Stage 3 exams are no longer administered in England. Between 1998 and 2013, children sat externally-marked English, mathematics and science exams and the scores from these tests are available in NPD.
KS4	14-16 (Years 10-11)	Includes GCSEs, GNVQs and other GCSE equivalents. Two files are available: an indicator file which contains an overall summary of each pupil’s main exam results and a results file which contains data on qualifications achieved by each pupil. Measures therefore include summaries such as whether a child achieved the English baccalaureate in addition to the subject-specific results. KS4 data are also available on children in private schools
KS5	16-18 (Years 12-13)	Children typically take the GCE, (also known as Advanced Subsidiary (AS) and Advanced (A) levels) before applying to university. Data on pupils in both schools and colleges are included and only students who complete two years of study (i.e. to Advanced level) are included. Data on individual subjects are available. Note that until 2013, participation in education beyond 16 in England was optional. Since then, children have a duty to remain in education or training until they are 17, and until 18 from 2015, though this need not necessarily be towards AS or A levels. KS5 data are also available on children in private schools.

### Social care data

In addition to information about children in schools, the NPD also has two modules on children looked after (for example in foster care) and children referred for local authority support (for example due to disability or for concerns about maltreatment). The latter dataset is often referred to as the children in need dataset or census though it also includes children who are assessed as not in need. Children are included in these datasets whether they are in school or not. If they are, they are linkable using the PMR. Both the children looked after and children in need datasets are subject to their own data resource profiles [14,15] and were studied as part of the Nuffield Family Justice Observatory data scoping study [16].

### Quantity and completeness

The number of children and schools recorded in each January census is given alongside Office for National Statistics (ONS) population estimates in Table 4. It is a legal requirement that all children receive education in England; for most children this will be in state schools from the age of five up to the age of 16. From 2013, children are required to be in some form of education or training until the age of 17 (the ‘participation age’), which was raised to 18 from 2015. Only children on state schools’ rolls are included in the NPD. Approximately 7% of all children in school each year are in independent (private) schools and an estimated 9% to 11% of children will have spent any time in a private school throughout their childhood, including Key Stage 5 (ages 16 to 18) at which stage participation in private education is significantly higher than at earlier stages [17]. Independent schools and general hospital schools do not participate in the School Census but instead provide data through the school level annual school census, which contains the numbers of pupils enrolled in these establishments but no child-level data. A number of children in England are home-schooled, with estimates in the range of 45,000 to 50,000 in 2017 [18,19] but no administrative data on them are available. The DfE ran a consultation on the registration and monitoring of home-schooled children between April and July 2018 [19].

**Table 4: Number of children of compulsory school age and number of children identified in the National Pupil Databases school censuses:** ONS Office for National Statistics. Sources: Department for Education [20] and ONS mid-year estimates [21]. * Only aggregate data are available for children in independent (private) schools.

	2011	2012	2013	2014	2015	2016	2017

ONS estimate, ages 5-15	6,680,298	6,701,384	6,736,870	6,795,108	6,872,599	6,970,434	7,100,054
*Number of pupils at January, ages 5-15*							
School Census	6,168,470	6,192,170	6,233,560	6,307,060	6,388,827	6,491,811	6,612,292
Pupil referral units	13,720	13,230	12,630	12,525	13,195	14,583	15,229
Alternative provision	18,305	17,670	17,790	15,555	15,551	16,149	16,640
Independent schools*	423,370	422,695	424,485	423,370	424,038	425,414	426,436
Total	6,623,865	6,645,765	6,688,465	6,758,510	6,841,611	6,947,957	7,070,597
% of all children	99.2%	99.2%	99.3%	99.5%	99.5%	99.7%	99.6%

## External linkage

### Linkage to publicly available information

Table 2 shows the school-level identifiers available. These are the unique reference number and establishment code (both unique identifiers for schools) and the LA-establishment number, which is the establishment number merged with the local authority identifying code. These identifiers can be used to link publicly available information, such as phase of education of the school (e.g. primary, secondary, middle or all-through) and type of school (e.g. free school, academy, special school), contained in the UK government’s ‘Get information about schools’ database (formerly EduBase). [22] Availability of the lower-layer super output area at child and school level also means that area-based indicators, such as the English indices of deprivation can be linked to children [23].

### Linkage to external record-level datasets

A number of other demographic variables which could facilitate linkage to external datasets such as the Hospital Episode Statistics (a longitudinal database of inpatient admissions, outpatient appointments and emergency department visits in England) are also available, including postcode, names, date of birth, age, gender and ethnicity (Table 2). For example, in a study on children aged five with isolated oral cleft, Fitzsimons et al [2] linked the NPD to the Hospital Episode Statistics and the Cleft Registry and Audit Network. They found that achievement on the Early Years Foundation Stage Profile in the children with oral cleft, especially with palate involvement, was significantly lower than the general population. Using national administrative data from health and the NPD enabled the researchers to evaluate early academic achievement as well as distinguish between different clinical presentations.

External linkage, however, is not straightforward owing to technical issues relating to data sharing and the optimisation of linkage algorithms and to governance requirements. In Fitzsimons et al [2], 89% of eligible children with a link from the Cleft Registry Audit Network to Hospital Episode Statistics subsequently linked to the NPD; it is not clear from the paper how the linkage algorithm affected match rates. Although administrative data are usually said not to suffer from attrition or selection biases, biases can nonetheless be introduced in linkage through false or missed links. Linkage between the NPD and mental health data in the Clinical Records Interactive Search—which consists of data from the South London and Maudsley National Health Service Foundation Trust—was assessed in depth by Downs et al. [24,25]. Details of the linkage methods, including the separation of person identifiable data and attributable data, are given in the paper. In brief, they involved standardisation of dates of births, names and addresses (using a hierarchical system for selecting the addresses most likely to match where multiple were recorded in the health and NPD data). A four-stage matching algorithm was then used, which included requirements of exact or fuzzy matches. The first stage achieved an overall match rate of 60.2% (of children in the health data to the NPD), followed by 4.2%, 1.2% and 16.9% (overall rate 82.5%). Some of these missed matches were likely due to private school enrolment or exclusive home schooling but the rest is unexplained. The authors were, however, able to evaluate associations between demographic and clinical characteristics and the odds of successful matching. They found, for example, a U-shaped relationship between the odds of matching and neighbourhood deprivation which they suggest may be due to (a) those in the most deprived neighbourhoods being more likely to need, and therefore attend, healthcare services; and (b) those in the least deprived neighbourhoods being better able to engage with administrative process and challenge and correct errors. A similar study evaluated linkage success using an opt-in consent model for Millennium Cohort Study participants and an opt-out consent model with the Clinical Records Interactive Search [26]. These examples show the importance of thoroughly investigating linkage algorithms for potential biases and the need to fully report all aspects of the linkage process: the Guidance for Information about Linking Data sets (GUILD) can be used in conjunction with traditional reporting checklists in this regard [27].

Because detailed demographic data are required (as opposed, for example, to a shared pseudonymised or anonymised identity code), projects involving such linkages will be considered on a case-by-case basis by data providers and researchers will have to ensure sufficiently stringent safeguards are in place for processing. As Downs et al show [25], this can take a number of years (almost four years to completion of linkage in their case), involving approvals from both DfE and the external bodies such as National Health Service research ethics committees, ensuring appropriate computer security and infrastructure is in place, consultation with service user groups and data preparation and linkage.

## Uses to date

### Monitoring trends

The NPD is used by the DfE to monitor school and pupil numbers and performance in England at each Key Stage. Extensive summary, aggregate data are made available by the DfE on an annual basis at national and local authority level [28]. These include annual data on exam results, absences and exclusions, with breakdowns given along various axes including gender, ethnicity, language and school type. Aside from giving snapshots of performance by different groups of children or schools, among other indicators, these data have been used in policy making processes. For example, the DfE statistics were referenced in a House of Common Education Committee inquiry report on alternative provision, off-rolling and exclusions [29] as well as in the government’s response [30].

### Research

Researchers have made extensive use of NPD data in a range of studies. A list of third-party data requests since April 2012 is available on the DfE website [31]. At the time of writing, there are approximately 480 listed projects. Examination of the list shows the range of studies being undertaken using NPD data on a wide range of educational issues such as: whether early school entry affects child behaviour relative to peers; the extent of pupil segregation; and evaluation of specific projects such as Sure Start Children’s Centres. Many examples are health-related, e.g., a Public Health England project on measuring the impact of influenza vaccination on school absence rates.

The NPD can also be used to remedy deficiencies in traditional study designs. It has been used to reduce bias attributable to missing outcome data in cohort studies [11] and in examining long-term outcomes in dormant randomised-controlled trials. The Head or Heart Study, for example, is linking data from dormant randomised-controlled trials, published between 1990 and 2009 on infant dietary enrichment, to the NPD in order to examine long-term associations between infant nutrition in those studies and school achievement [32]. Although some long-term follow up work has been conducted with these participants, attrition increases in older children, potentially biasing results. Linking to the NPD means that participants who are lost to follow up can be still be included, thereby overcoming attrition bias. This should enable evaluation of the long-term cognitive effects of dietary supplements in infancy [32].

### Longitudinal analysis

A particular strength of the NPD is the longitudinal linkage of events over the child life course. Whereas cross-sectional analyses provide an estimate of population burden of given exposures at a given point in time, only longitudinal analyses provide a cumulative estimate and therefore a fuller appraisal of burden. Additionally, accounting for the time-varying nature of many exposures can provide a more fine-grained analysis that more accurately assesses exposure-outcome relationships. However, longitudinal analyses with administrative data poses difficulties due to changes over time in real-world practice, coding, and availability of datasets. For example, the GCSE marking recently changed in 2017 from a system with grades A* to U to a new system with grades 9 to 1 for English and mathematics (with other subjects changing in 2018 and 2019). The two systems are not directly comparable. There may also be changes in coding that do not reflect changes to practice (e.g. the ‘Date of joining’ was renamed ‘Entry date’ in 2002/3; ethnicity was coded numerically in 2001/2, which was changed to text codes from 2002/3 onwards) and some variables are discontinued (e.g. ‘Mode of travel’ was only available between 2006/7 and 2010/11). Finally, as Figure 1 shows, entire datasets may become obsolete (e.g. Key Stage 3 tests are no longer administered and therefore no longer form part of the NPD) or merged (e.g. the Pupil Referral Unit census was merged with the School Census in 2013/14). Longitudinal analysis requires mapping of changing school identities over time. A database that tracks school mergers, splits, closures, openings and changes in status from 1999 to 2014 is available from the UCL Institute of Education via the Cohort and Longitudinal Studies Enhancement Resources [33].

### NPD user group

The Centre for Market and Public Organisation at the University of Bristol hosts the NPD User Group, which holds an annual meeting for those with interests in NPD data. NPD users can join the mailing list detailed on the User Group website as well as inspect slides from previous meetings [34]. The group offers a forum for data users to meet to discuss working with the NPD. Attendees have included representatives from the DfE, academia, the Universities and Colleges Admissions Service, the Greater London Authority and independent organisations such as the National Foundation for Educational Research.

## Access and governance

Timely access to administrative data resources is of crucial importance to ensure effective service evaluation and redesign. [35] The DfE is publicly committed to sharing data for research [8,36] and has for years provided free access to NPD data for approved research projects. However, researchers must be aware that the process from initial study design to data access can consume significant amounts of time ranging from months for simple requests involving only NPD data to years for complex cross-sectoral linkage projects, as noted above.

The DfE’s website provides information on how to request access to data [8]. Requests will be considered by the DfE’s Data Sharing Approval Panel (formerly Data Management Advisory Panel) who will ensure the project is safe (using the ‘Five Safes’ framework [5]), that researchers hold a Disclosure and Barring Service certificate (i.e. a police records check) and that relevant declarations have been signed by the researchers. Details on membership, which consists of DfE and non-DfE members, working practice and terms of reference of the Panel are available on the DfE’s website [5]. Those requesting access to data will be required to demonstrate how their project has public benefit and they will be asked to specify individual variables requested (though the DfE has indicated that standard extracts are also available [8]). Details on the lawful bases of data collection and sharing are available on the DfE’s website [5].

Recognising the resource constraints of the DfE, concerns around data security and breach risks and new requirements under the General Data Protection Regulation, the DfE between May and September 2018 developed and instigated a new data access process in conjunction with the ONS. If access is granted, most extracts will be made available through the ONS Secure Research Service network of secure labs or secure online environment. [37] As such, researchers wishing to access NPD data are required to have ONS Approved Researcher accreditation. [38] Access to NPD data through a single safe haven may introduce an added layer of complexity for cross-sectoral linkage and such projects will have to be negotiated on a case-by-case basis; the DfE have stated that other arrangements may be possible subject to computer and physical security [5]. Alongside these developments, the DfE has also updated privacy notices for schools and local authorities that are designed to provide information on the NPD and its uses to pupils, parents and others [39]. Finally, the DfE has stated that it also plans to develop an online application procedure, more standard extracts, and publicly available synthetic data to help test and plan research [8].

Adhering to such safeguards and governance processes imposes a burden on researchers but is important for maintaining public support in the sharing of data for research. In December 2018, two of the authors (MAJ and RG) carried out a public engagement session with a group of Young Research Advisors (ten young people aged 9 to 24 years from primary school to post-university) facilitated by the National Children’s Bureau on the topic of data sharing involving the NPD and health data. Participants were supportive of anonymous data sharing for research (including the sharing of identifiers for linkage through a third party model) providing sufficient safeguards are in place. The group also recognised the potential benefits of sharing data between services to support individual decision-making. However, the need for individual consent was highlighted, as was the suggestion that data flows from health to education would require more stringent safeguards, rather than the other way around. This highlights the need to consider carefully the direction of data flows in whether a linkage project is likely to obtain public support.

## Strengths and limitations

A major strength of the NPD lies in its near whole population coverage (Table 4). It is not subject to participant non-response or attrition and large numbers admit sufficient power to support analyses of rare as well as common exposures and outcomes. Linkage of children across census years and different Key Stages also enables longitudinal analysis of factors such as school attendance and exclusion, mobility and attainment. As noted above, the rich set of demographic variables facilitates linkage to third-sector data resources. Finally, routinely published aggregate data are available from the DfE [28].

Key limitations include exclusion of children in private schools and those who are home schooled (see ‘Quantity and completeness’). Additionally, as discussed under ‘Uses to date,’ longitudinal analyses are also challenging as a result of changes over time in dataset availability, coding, and real-world practice. We are unaware of any databases that provide a systematic overview of publications using NPD data. The DfE’s list of approved projects contains structured data only on access process matters (e.g. licence start and end dates and tier of data approved) and not subject matter [31]. The creation of a searchable and thematic database of projects and publications would enable easier identification of the types of uses of NPD data, the resulting publications and the impact that they have.

### Future directions and conclusions

As noted above, the DfE is publicly committed to continue to provide access to NPD data for approved research projects and a number of linkage and research projects are underway using the NPD. The Economic and Social Research Council’s Administrative Data Research Partnership is ‘an investment in research infrastructure to maximise the potential of administrative data as a resource for high-quality research in the UK’[40]. It aims to make use of existing administrative data resources and new provisions in the Digital Economy Act 2017 that enable and facilitate cross-sectoral data linkage for research purposes. One of the Partnership’s priorities is to link NPD data to the 2011 census (i.e. the national census) in order to obtain household characteristics, which are absent from the NPD (Ruth Gilbert, personal communication). Harron et al [41] conducted a scoping report into the use of linked administrative data to evaluate the Family Nurse Partnership—an intervention aimed at supporting young parents and recommended, among other things, that mother-child linkage using Hospital Episode Statistics be carried out, as well as linkage to NPD to evaluate relevant developmental indicators. Such a project has been approved and funded and is about to start (Ruth Gilbert, personal communication). Work is also under development to build on studies examining education and physical and mental health [2,24,25].

The NPD is an especially valuable data resource for researchers interested in the educational experience and outcomes of children and young people in England. Although limited by the fact that children in private schools or home schooled are not captured in the censuses, it provides a near-complete picture of school trajectories and outcomes for the majority of children. Linkage to other datasets can enhance analyses and provide answers to questions that would otherwise be extremely difficult to find.

## Abbreviations

DfE	Department for Education
GCSE	General Certificate of Secondary Education
NPD	National Pupil Database
ONS	Office for National Statistics
PMR	Pupil Matching Reference
